# Retreatment With Nivolumab and Ipilimumab in Pleural Mesothelioma Following Disease Progression After a Durable Response: Case Series

**DOI:** 10.1016/j.jtocrr.2025.100835

**Published:** 2025-04-21

**Authors:** Illaa Smesseim, Paul Baas, Jacobus A. Burgers

**Affiliations:** a Department of Thoracic Oncology, Netherlands Cancer Institute, Amsterdam, The Netherlands; b Department of Pulmonology, Leiden University Medical Center, Leiden, The Netherlands

**Keywords:** Immunotherapy, Mesothelioma, Retreatment, CheckMate 743, Nivolumab, Ipilimumab

## Abstract

The CheckMate 743 trial established nivolumab and ipilimumab as the standard first-line treatment for unresectable pleural mesothelioma. However, optimal management following disease progression after a durable response to dual immunotherapy remains unclear. We report two cases of patients with pleural mesothelioma (epithelioid subtype) initially treated with nivolumab-ipilimumab, achieving prolonged disease control. Both patients experienced disease progression several years after treatment discontinuation and were subsequently retreated with nivolumab-ipilimumab on regulatory approval. In both cases, retreatment resulted in stable disease for at least 12 months. However, immune-related toxicities occurred, with one patient developing recurrent colitis and the other experiencing nephrotic syndrome, ultimately leading to treatment discontinuation. These cases suggest that retreatment with dual immunotherapy may be a viable strategy for selected patients with previous durable responses, although the risk of immune-related toxicity remains significant. Given the lack of prospective data, further research is needed to determine whether rechallenge with nivolumab-ipilimumab offers superior outcomes compared with chemotherapy or best supportive care in this setting. Rechallenging patients with pleural mesothelioma with nivolumab-ipilimumab after a durable response is feasible but associated with immune-related toxicity.

## Introduction

The CheckMate 743 trial revealed significantly improved overall survival in adult patients with untreated, unresectable pleural mesothelioma treated with first-line nivolumab (a programmed cell death protein 1 inhibitor) and ipilimumab (a CTLA-4 inhibitor) for up to two years.^1^ Clinical guidelines now recommend dual immunotherapy as the standard first-line treatment for these patients.^2^^,^^3^ No published studies to date have specifically investigated second-line treatments for patients who progressed after dual immunotherapy or patients who experienced disease progression after a durable response after the completion of treatment with nivolumab-ipilimumab. For patients with an Eastern Cooperative Oncology Group performance status score of 0 to 2 who experienced disease progression after dual immunotherapy, guidelines recommend a platinum-pemetrexed regimen as second-line therapy. For those with a poor performance status (Eastern Cooperative Oncology Group > 3), best supportive care remains the advised approach. The ongoing PEMMELA 2 study, a phase 2 trial, is investigating the objective response rate of combining pembrolizumab and lenvatinib in patients with pleural mesothelioma previously treated with nivolumab and ipilimumab.^4^

The question remains about the optimal second-line management of patients with pleural mesothelioma who experienced durable disease response to dual immunotherapy and whether retreatment with dual immunotherapy is a viable option. Data discussing rechallenge of the same immunotherapy after discontinuation of treatment are all retrospective in nature and not specific for pleural mesothelioma. For example, a previous study revealed that patients with melanoma who were retreated with immunotherapy after previous disease control had a high disease control rate (75%).^5^ In this case series, we describe two patients who were treated with first-line nivolumab and ipilimumab in the INITIATE study and achieved a prolonged disease response. On disease progression, they were treated again with nivolumab and ipilimumab.

## Case Presentations

### Case 1

**Figure 1:**
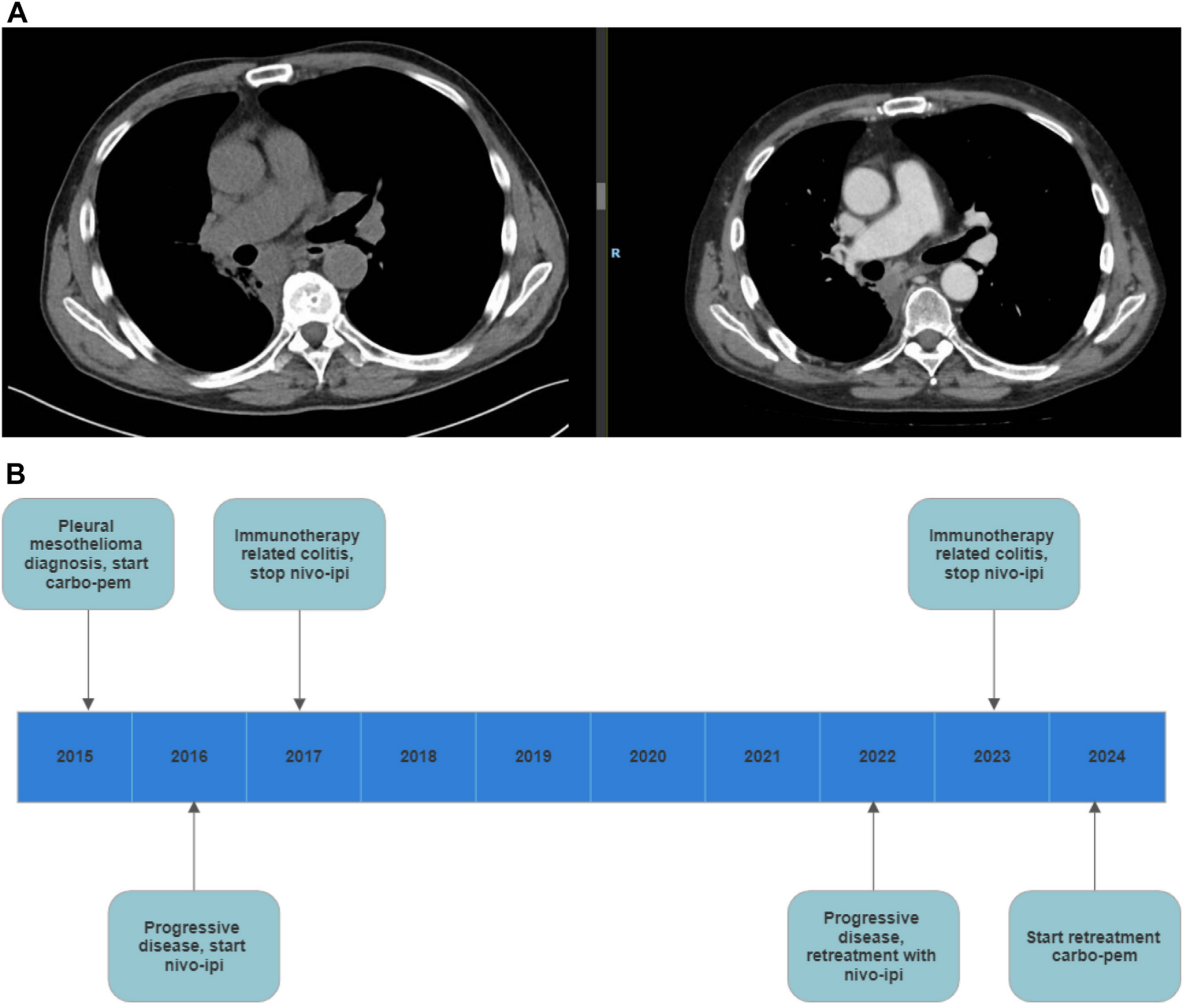
(*A*) Case 1: CT scan (without contrast) 2 weeks before start of retreatment with nivo-ipi (left) and CT scan (with contrast) 9 weeks after start of retreatment with nivo-ipi (right). (*B*) Case 2: Timeline of events. Carbo-pem, carboplatin-pemetrexed; CT, computed tomography; nivo-ipi, nivolumab-ipilimumab.

A 66-year-old patient diagnosed with right-sided pleural mesothelioma (epithelioid subtype, programmed death-ligand 1 unknown) was referred to our hospital for treatment 1 year after disease progression following first-line carboplatin-pemetrexed. The patient was enrolled in the INITIATE study and treated with nivolumab and ipilimumab.^6^ Although a partial response was achieved with this treatment according to Response Evaluation Criteria in Solid Tumors (RECIST) 1.1, immunotherapy had to be discontinued after 6 months due to a Common Terminology Criteria for Adverse Events (CTCAE) grade 3 immunotherapy-related colitis. This was managed with corticosteroids (60 mg prednisolone per day, tapered to zero after 8 weeks). The disease remained stable for 5 years. Unfortunately, 5 years after discontinuation of treatment, a new lesion was detected on a computed tomography (CT) scan. The option of starting second-line chemotherapy was discussed with the patient. However, because he had previously experienced a durable 5-year response to dual immunotherapy, he wished to explore whether restarting dual immunotherapy was an option. A request was submitted to Bristol-Myers Squibb for retreatment with immunotherapy, which was approved. Restarting dual immunotherapy again resulted in stable disease. However, after 1 year of treatment, the patient again developed a CTCAE grade 3 immunotherapy-related colitis, necessitating treatment with corticosteroids (60 mg prednisolone per day, tapered to zero after 10 weeks) and discontinuation of dual immunotherapy. As colitis symptoms recurred during corticosteroid tapering, the decision was made to discontinue immunotherapy. The disease remained stable for more than a year. Unfortunately, follow-up scan results revealed progressive disease according to RECIST 1.1, and rechallenge treatment with carboplatin-pemetrexed was discussed with the patient and he is currently being treated with chemotherapy (Fig. 1*A* and *B*).

### Case 2

**Figure 2:**
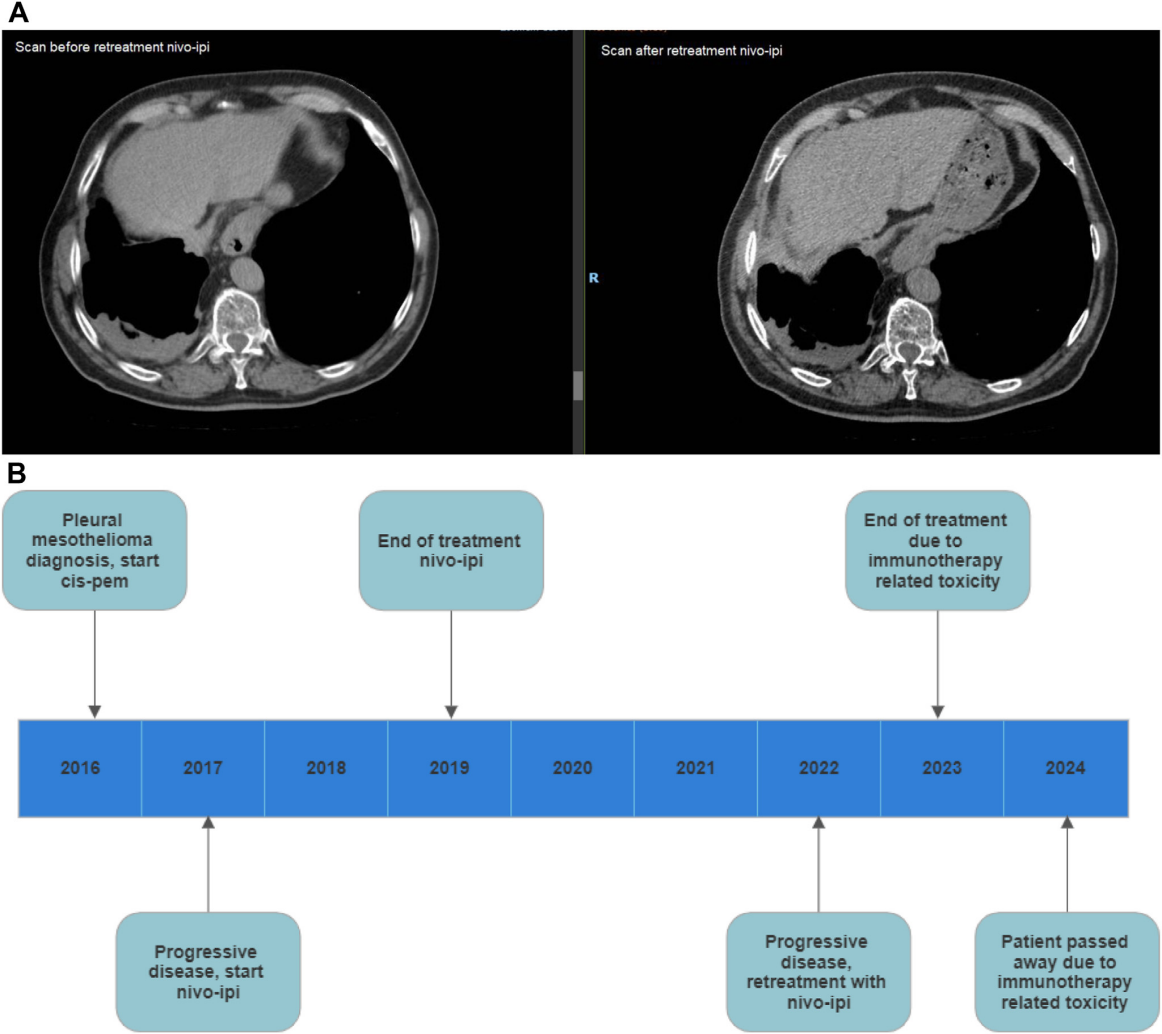
(*A*) CT scan (without contrast) 1 week before start of retreatment with nivo-ipi (left) and CT scan (without contrast) 6 weeks after start of retreatment with nivo-ipi (right). (*B*) Timeline of events. Cis-pem, cisplatin-pemetrexed; CT, computed tomography; nivo-ipi, nivolumab-ipilimumab.

An 82-year-old patient diagnosed with pleural mesothelioma (epithelioid subtype, programmed death-ligand 1: 30%) was initially treated with four cycles of carboplatin-pemetrexed. Unfortunately, just 3 months after treatment with first-line chemotherapy, disease progression was observed on a CT scan. The patient was then treated with second-line nivolumab and ipilimumab as part of the INITIATE study.^6^ Follow-up scan results revealed a partial response, and the treatment was well tolerated. After 2 years of therapy, dual immunotherapy was discontinued (end of treatment). The patient had no immunotherapy-related toxicity during treatment. After 3 years of follow-up, disease progression was again detected on a CT scan, and the patient developed symptoms of CTCAE grade 2 dyspnea. Given the patient’s durable response to previous dual immunotherapy, he opted to explore whether restarting dual immunotherapy was an option, and a request was submitted to Bristol-Myers Squibb for rechallenge with nivolumab-ipilimumab. This request was approved, and the patient was subsequently treated with nivolumab and ipilimumab for another 1.5 years, resulting in stable disease according to RECIST 1.1. However, treatment had to be discontinued due to CTCAE grade 3 immunotherapy-related nephrotic syndrome, which was treated with corticosteroids (60 mg prednisolone per day tapered to 5 mg per day after 10 weeks). Unfortunately, the patient’s clinical condition deteriorated with CTCAE grade 3 dyspnea and weight loss, and he passed away after a few months (Fig. 2*A* and *B*).

## Discussion

These cases describe two patients with pleural mesothelioma (epithelioid subtype) with a durable response of more than 3 years after discontinuation of nivolumab-ipilimumab treatment who were treated again with dual immunotherapy after disease progression. In both cases, no further tumor growth was observed for at least 12 months. Because of the small sample size, the findings of this case series cannot be generalized to a broader population. However, these cases suggest that retreatment with nivolumab-ipilimumab may be a promising option for patients with pleural mesothelioma who previously achieved a durable response with this treatment.

It was also revealed that retreatment of nivolumab-ipilimumab carries a risk of immunotherapy-related toxicities. Data from patients with NSCLC indicate that restarting the same immunotherapy carries a 50% to 71% risk of developing immune-related toxicities. Notably, 50% to 60% of these patients experience a recurrence of the same type of toxicity.^7^, ^8^, ^9^ Our first patient experienced grade 3 immune-related colitis during his initial treatment with nivolumab-ipilimumab, which necessitated discontinuation of the therapy. Unfortunately, the same toxicity recurred when the treatment was restarted. The second patient, who did not experience immune-related toxicities during his initial treatment with nivolumab-ipilimumab, developed CTCAE grade 3 immune-related nephrotic syndrome after the therapy was restarted. This suggests that retreatment with the same immunotherapy regimen carries a risk of developing immune-related toxicity, even if it was not experienced during the initial treatment.

Furthermore, it is important to note that, although we chose to treat these patients with immunotherapy retreatment, we cannot rule out the possibility that they might have also experienced a positive response to treatment if they had both received chemotherapy instead. To date, no data have been published on patients with pleural mesothelioma who restarted the same immunotherapy regimen after completing treatment with nivolumab-ipilimumab.

Because nivolumab-ipilimumab is now recommended as a first-line treatment in the guidelines, we anticipate an increasing need for guidance on how to treat patients who become progressive after achieving a prolonged disease response. Future research should focus on evaluating whether restarting the same immunotherapy regimen is a viable and safe option.

## Conclusion

Rechallenging with nivolumab-ipilimumab in patients with pleural mesothelioma may be a viable option, though it carries a risk of immune-related toxicity. Further research is needed to determine whether restarting dual immunotherapy is more effective than chemotherapy or best supportive care.

## CRediT Authorship Contribution Statement

**Illaa Smesseim:** Conceptualization, Methodology, Formal analysis, Investigation, Resources, Data Curation, Writing - original draft, Visualization, Project administration.

**Paul Baas:** Investigation, Resources, Data Curation, Writing - original draft, Visualization, Supervision.

**Jacobus A. Burgers:** Investigation, Resources, Data Curation, Writing - original draft, Visualization, Supervision.

## Disclosure

Dr. Baas has received research grants and travel support and is consultant for Bristol Myers Squibb and 10.13039/100009947Merck Sharp & Dohme for his institution. Dr. Burgers received funding of an investigator-initiated study by Merck Sharp & Dohme. Ms. Smesseim has nothing to declare.
